# Spatiotemporal distribution and determinants of delayed first antenatal care visit among reproductive age women in Ethiopia: a spatial and multilevel analysis

**DOI:** 10.1186/s12889-021-11614-x

**Published:** 2021-08-19

**Authors:** Daniel Gashaneh Belay, Fantu Mamo Aragaw, Denekew Tenaw Anley, Yibeltal Shitu Tegegne, Kassahun Alemu Gelaye, Zemenu Tadesse Tessema

**Affiliations:** 1 Department of Human Anatomy, College of Medicine and Health Sciences, University of Gondar, Gondar, Ethiopia; 2 Department of Epidemiology and Biostatistics, Institute of Public Health, College of Medicine and Health Sciences, University of Gondar, Gondar, Ethiopia; 3 Department of Epidemiology and Biostatistics, School of Public Health, College of Medicine and Health Sciences, Debretabor University, Debretabor, Ethiopia; 4 Department Of Epidemiology Curative and Preventive Health Service, Amhara Regional Health Bureau, Bahir Dar, Ethiopia

**Keywords:** Delayed first ANC visit, Multilevel analysis, Spatiotemporal distribution, Ethiopia

## Abstract

**Background:**

Antenatal care (ANC) is one of the four pillars of the initiative for safe motherhood. ANC helps to improve the health of pregnant women and reduce the risk of adverse pregnancy outcome. First ANC is used to know the health status of the mothers and the fetus, to estimate the gestational age and expected date of delivery. Our research aims to investigate the Spatio-temporal distribution of delayed first ANC visit and its predictors using multilevel binary logistic regression analysis.

**Method:**

A total of 10,184 women (2061 in 2005, 3366 in 2011, and 4757 in 2016) were included for this study. The data were cleaned and weighted using STATA version 14. A multilevel binary logistic regression model was fitted to identify significant predictors of delayed first ANC visit. ArcGIS software was used to explore the spatial distribution of delayed first ANC visits and a Bernoulli model was fitted using SaTScan software to identify significant clusters of delayed first ANC visits.

**Results:**

Overall, 77.69, 73.95, and 67.61% of women had delayed their first ANC visit in 2005, 2011, and 2016 EDHSs respectively. Women education [AOR = 0.71; 95%CI; 0.60, 0.84], unwanted pregnancy [AOR = 1.41;95%CI; 1.04, 1.89], and rural residence [AOR = 1.68;95%CI; 1.19, 2.38] have significantly associated with delayed first ANC visit. The spatial analysis revealed that delayed first ANC visit varies in each EDHS period. The SaTScan analysis result of EDHS 2005 data identified 122 primary clusters located between the border of Oromia and Eastern SNNPR regions (RR = 1.30, LLR = 32.31, *P*-value< 0.001), whereas in 2011 EDHS, 145 primary clusters were identified in entire Tigray, B/Gumuz, Amhara western part of Afar and northwest Oromia regions (RR = 1.30, LLR = 40.79, *P*-value< 0.001). Besides in 2016 EDHS,198 primary clusters were located in the entire SNNPR, Gambella, Northen B/Gumuz, and western Oromia regions. (RR = 1.35, LLR = 83.21, *P*-value< 0.001).

**Conclusion:**

In Ethiopia delayed first ANC visit was significantly varied across the country over time Women’s education, wanted the last child, and residence were significantly associated with delayed first ANC booking. The effect of each predictor was found to be different across regions of Ethiopia. Therefore, a targeted intervention program is required in highly affected areas of Ethiopia.

## Background

One of World Healh Organization (WHO’s) top priorities is to promote maternal health, including antenatal care (ANC), which is one of the Healthy Motherhood Initiative’s four pillars [1]. Maternal health refers to the well-being of women before, during, and after pregnancy, as well as during childbirth and the postpartum period [2]. Antenatal care (ANC) can be defined as the care provided by skilled healthcare providers to pregnant women and adolescent girls to ensure the best maternal and child health during pregnancy [3]. Antenatal maternity care is a way for pregnant women to enter the health-care system and allows them to arrange the resources they need to have a stable pregnancy, a safe childbirth, and a healthy mother-baby pair [1, 4].

The ANC promotes the health of pregnant women and has been demonstrated to reduce the risk of unfavorable pregnancy effects, perinatal and fetal mortality, and morbidity [5, 6]. The ANC’s early visit allows health care workers to test and treat numerous maternal and fetal health issues as soon as possible, such as malnutrition, sexually transmitted diseases, congenital malformations, and other pregnancy complications [5]. The first stair flight to hit the maximum of success for healthy motherhood is antenatal care (ANC) [6]. For early detection of pregnancy-related problems and unfavorable pregnancy outcomes, like low birth weight, stillbirth, intrauterine fetal death and other complications, early initiation of ANC are essential, however, most of the mothers initiate ANC late [7, 8].

Prenatal care is more likely to be successful if women start receiving care in the first trimester of pregnancy and continue to receive care throught pregnancy, according to agreed guidelines [9]. The first antenatal check-up is defined as the first time a pregnant woman attends to a maternity health services to receive health care. It is used to consider the health status of mothers and the fetus, to predict the gestational age (GA) and expected delivery date (EDD), and to launch plans for future follow-ups [7].

The first trimester of pregnancy is the fetus’s most rapid developing stage, during which all of its organs are fully developed and require special care [10]. ANC follow-up during this period indirectly saves the lives of babies and mothers by promoting and establishing good health and proper nutrition of both the child [11]. Malnutrition of the child in the first 1000 days which results from poor adherence to ANC is associated with poorer cognitive development and lower educational outcomes [12].

Pregnant women in developing countries should have at least four ANC checks, with the first one occurring before the gestational age of 12 weeks, according to the World Health Organization (WHO) [13].

Daily, 830 women die in the world as a result of pregnancy and childbirth-related problems, and more than 303,000 women die annually [14]. Almost all, of those deaths, occur in developing countries, and from this, Sub-saharan Africa alone accounts for about 66%, followed by Southern Asia 22% [14]. Furthermore, it is reported that 2.6 million babies are stillborn, with another 4 million newborns dying during the neonatal period. Many of these deaths may have been prevented without delay during the first visit by measures offered as part of basic antenatal care services [14, 15].

While the worldwide coverage of early prenatal care visits rose by 43% between 1990 and 2013, only less than half of all women in less developed countries had early prenatal care visits [16]. Maternal mortality continues to vary between developing and developed countries. The overall lifetime risk of a woman’s death due to pregnancy and related causes is estimated at 1 in 180 in developing countries, while it is around 1 in 4900 in developed countries [17].

Ethiopia is one of the Subsaharan African countries which have a high magnitude maternal mortality rate which was 401 per 100,000 live births in 2017 and remains among the highest in the world [10, 18]. In developing countries like Ethiopia, obstetric complications during pregnancy and childbirth are the leading causes of death among reproductive-aged women [19, 20]. The lack of access and insufficient use of antenatal care (ANC) during pregnancy is widely recognized as leading to adverse maternal health outcomes. Antenatal care uptake is one of the key indicators for monitoring the progress of improving maternal outcomes [8, 19–21].

A systematic study and meta-analysis conducted in Ethiopia found that the pooled magnitude of delayed ANC was 64% [17]. In another study in Ethiopia also showed 89% of women made their first antenatal care visit after the fourth month of pregnancy nationwide [10]. The study was conducted based on the 2011 Ethiopian demographic and health survey (EDHS) and, a study conducted in Addis Zemen primary hospital showed that 66.3 and 52.5% of women did not use ANC in the first trimester respectively [22, 23]. But in the study conducted in the Tigray region, Ethiopia it was 27.5% [24].

**Fig. 1:**
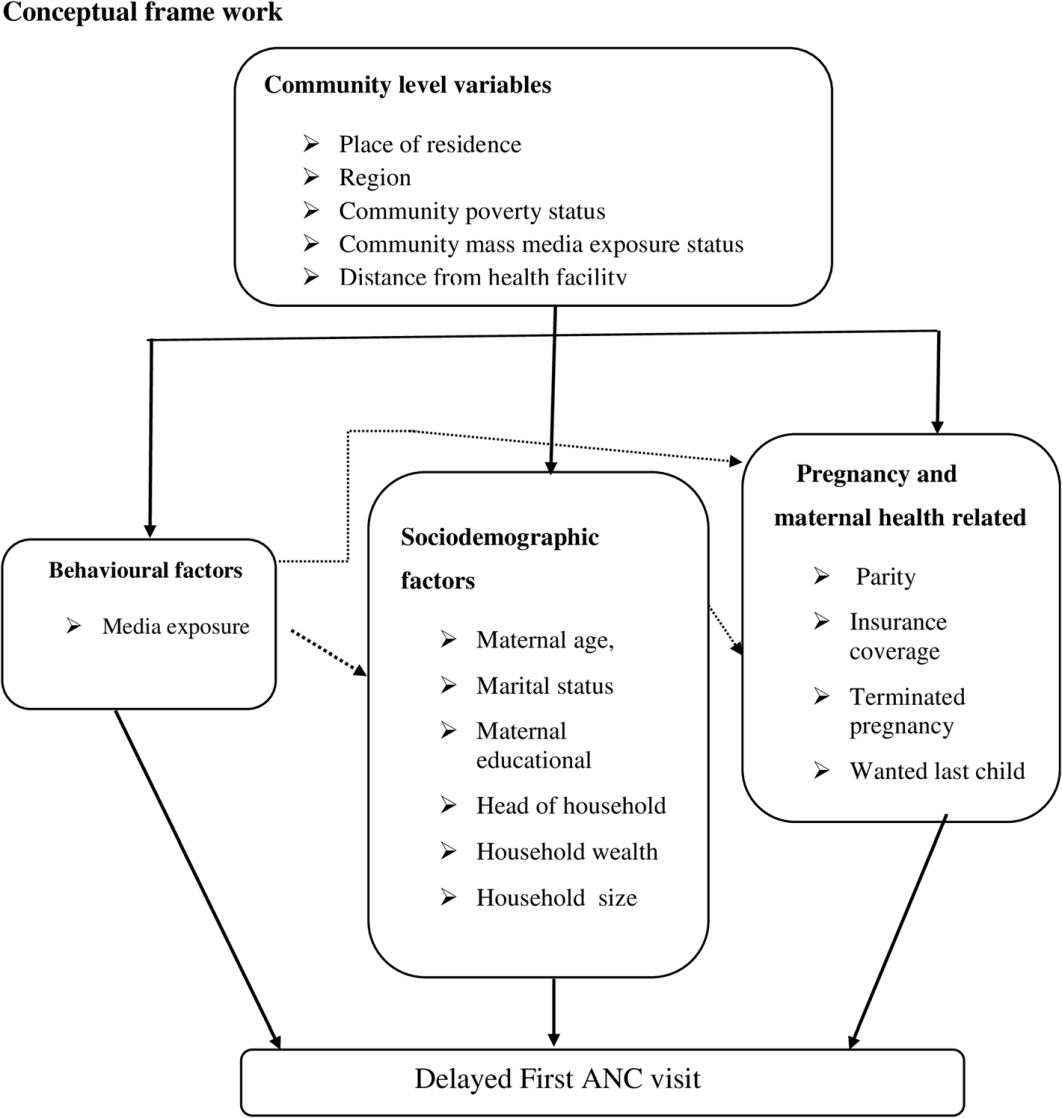
Conceptual framework of delayed first ANC visit [4, 24]

Different studies on determinants of antenatal care utilization of show that women living in urban residence [5], having old age [5], having low parity [5], being educated and having an educated partner [5, 24], being employed [5, 22], being exposed to mass media [5], being married [5, 25], and having an ANC follow up at a private hospital or clinic [25] had a positively associated with good ANC attendance and timeliness (Fig. 1).

However, having an unplanned pregnancy [5, 22, 24], having previous pregnancy complications [5], having more than six children [25], lack of husband’s support [5], increased distance to the health facility [5, 22, 25], and not having health insurance and high cost of services [5, 25], were found to be the cause for delayed antenatal visits (Fig. 1).

This study hypothesized that there is a variation in the distribution of delayed first ANC visits in time and region in Ethiopia and there are factors that contribute to these variations. Therefore this study aimed to investigate the spatiotemporal distribution of delayed first ANC visits and its predictors using multilevel binary logistic regression analysis which could certainly provide new evidence helpful for implementers for highly targeted and effective interventions.

The findings of this study are also essential in determining which factors are most important in postponing their first ANC visit, as well as answering the question of when and where delays in their first ANC visit are more common, and what are the causes for this spatiotemporal distribution.

## Methods

### Study design, setting, and period

A cross-sectional survey study design was conducted based on 2005, 2011, and 2016 EDHS. Ethiopia is an East African country with a population of 100,613,986 people, making it the second most populous country in Africa. Administratively, Ethiopia is decentralized at the federal level, subdivided into nine regions and two city administrations and regions are divided into zones, and zones, into administrative units called Woredas. Each Woreda is further subdivided into Kebeles, which are the smallest administrative units. Kebele is also subdivided into census enumeration areas (EAs), which are convenient for the implementation of census. The detail of the study design and setting are detailed elsewhere in Central Statistical Agency (CSA) Ethiopia [26].

### Source and study population

All reproductive age women (15–49) in Ethiopia were the source population. The study population consisted of women who had given birth in the 5 years before to each survey and had ANC follow-up for the most recent birth. The study excluded women who did not have ANC follow-up after a recent birth, women who did not have birth records, and clusters with zero coordinates.

### Sample size and sampling procedure

The poll was conducted in Ethiopia’s nine regions and two city administrations. In each survey year, participants were selected using a stratified two-stage cluster sampling technique (2005, 2011, and 2016). After excluding no birth records and clusters with zero coordinate, a total of 1720 clusters (527 clusters in 2005, 571 clusters in 2011, and 622 clusters in 2016) were included. The detailed sampling procedure was included in each EDHS reports from the Measure DHS website (www.dhsprogram.com). Weighted values were used to maintain the representativeness of the sample data and calculated from children’s record (KR) EDHS datasets. Finally, this study included a total weighted sample of 10,184 women (2061 in 2005, 3366 in 2011, and 4757 in 2016) was included for this study.

### Outcome variable

The outcome variable of this study was delayed first ANC visit which means mothers who have first ANC booking after 12 weeks of gestational age [27] which have a binary response. A woman said having delayed first ANC visit if she books her first ANC visit after 12 weeks of pregnancy and coded as “1”, and women said having early first ANC visit if she books her first ANC before 12 weeks gestation and coded it as “0.” [4, 19].

### Independent variables

Independent variables at the individual and community levels were considered. Individual socio-demographic variables such as maternal age, marital status, maternal education, household size, household head, and household wealth were included in the individual-level factors. Characteristics of pregnancy and maternal health, such as parity,ever had terminated pregnancy, insurance coverage, and pregnancy desirability were also considered. Finally, behavioral characteristic like media exposure were included. Media exposure status is created from the frequency of reading a newspaper or magazine, watching TV, and listening to the radio. If a woman has at least one yes, she has considered to have media exposure. The community-level factors include the place of residence, region, perception of distance from the health facility, community-level media exposure, and community level poverty were considered. The level of poverty in the community was determined by the proportion of women in the poorest and poorest quintiles obtained from the wealth Index results. It was coded as “0” for low (communities in which < 50% women had poor and poorest wealth quintiles), “1” for high (communities in which ≥50% women had poorest and poorer wealth quintiles) poverty communities. Community-level media exposure was assessed by the proportion of women who had at least been exposed to one media. It employs``` “0” to indicate low-level and “1” to indicate high-level media coverage at the community level [4, 28].

### Data management and analysis

After obtaining permission via an online request explaining our research purpose, this study was conducted using data from the three EDHSs received from the official DHS measure website www.measuredhs.com. We extracted the outcome and independent variables from the collection of Child data (KR) data [26]. Based on the Guide to DHS Statistics in Microsoft Excel and STATA version 14, data was cleaned and recoded. Before conducting any statistical analysis, we weighted for sample probabilities and non-response using the weighting factor provided in the EDHS data, as per the survey report’s suggestion, to restore the survey’s representativeness and obtain valid statistical estimates. The weighted proportion of delayed first ANC visit data were exported to ArcGIS. Then, for spatial distribution, spatial autocorrelation, incremental autocorrelation, spatial interpolation, and detection of hot spot areas, Arc GIS 10.7 software was used.

### Model building

In multi level analysis four models were fitted. The first was the null model (Model 1) used to check the variability of delayed first ANC visits in the community and only containing the outcome variables. The second (model 2) and third (model 3) hierarchical models contain individual-level variables and community-level variables, respectively. In the fourth model (Model 4) both community and individual level variables with the delayed first ANC visit were fitted simultaneously.

### Parameter estimation method

Fixed effects (a measure of association) are used to assess the relationship between likelihood of delayed first ANC visit and explanatory variables at both individual and community levels. Factors with a *p*-value ≤0.2 in crude odds ratio (COR) were selected as candidates for the adjusted model. Finally the ssociations between dependent and independent variables were assessed and its strength was presented with adjusted odds ratios and 95% confidence intervals with a *p*-value of < 0.05.

**Random-effects (a measure of variation)** were estimated by the median odds ratio (MOR), ICC, and Proportional Change in Variance (PCV).

MOR is defined as the central value of the odds ratio between regions highest risk and the lowest risk when randomly picking out two clusters.

MOR = exp.[√(2 × VA) × 0.6745], or $\kern1.00em \mathrm{MOR}={e^{0.95}}^{\sqrt{VA}}$ where; VA is the area level variance [28–30].

The PCV reveals the variation in delayed first ANC visits among reproductive-age women explained by factors. The PCV is calculated as; $PCV=\frac{Vnull- VA}{V\  null}\ast 100\%$ where; Vnull = variance of the initial model, and VA = variance of the model with more terms.

The ICC which reveals the variation of delayed first ANC visit between clusters is calculated as; $ICC=\frac{VA}{VA+3.29}\ast 100\%$, where; VA = area/cluster level variance [28–30].

### Spatial distribution and autocorrelation

Spatial autocorrelation (Global Moran’s I) statistic measure was used to assess whether delayed first ANC visit was dispersed, clustered, or randomly distributed in Ethiopia [31].

### Hot spot analysis

For hotspot analysis, the proportion of delayed initial ANC visits in each cluster was used as an input. Hot Spot Analysis of the z-scores and significant *p*-values (Getis-Ord Gi* statistic) identifies locations with hot or cool spot values concentrated in space. The hot spot areas indicated that there was a high proportion of delayed first ANC visits, while there was a low proportion in the cool spot areas.

### Spatial interpolation

We used a geostatistical ordinary Kriging spatial interpolation technique using ArcGIS 10.7 software for predict delayed first ANC visits to unsampled areas based on sampled clusters.

### Spatial scan statistics

The geographic location of statistically significant clusters for delayed first ANC visit was determine using Kuldorff’s SaTScan version 9.6 software [32]. The scanning window that moves across the study area in which women had delayed the first ANC visit were taken as cases and those women who had early first ANC visit taken as controls to fit the Bernoulli model. Using *p*-value and likelihood ratio tests the most probable cluster were determined based on 999 Monte Carlo replicates.

### Ethical consideration

After describing the objectiveof the analysis, the data sets were downloaded with permission from the Measure DHS website http://www.dhsprogram.com.

No personal names or household addresses are in the data collection. The information was used specifically for the registered study topic and was not shared with any person.

## Results

### Background characteristics of study subjects

**Fig. 2:**
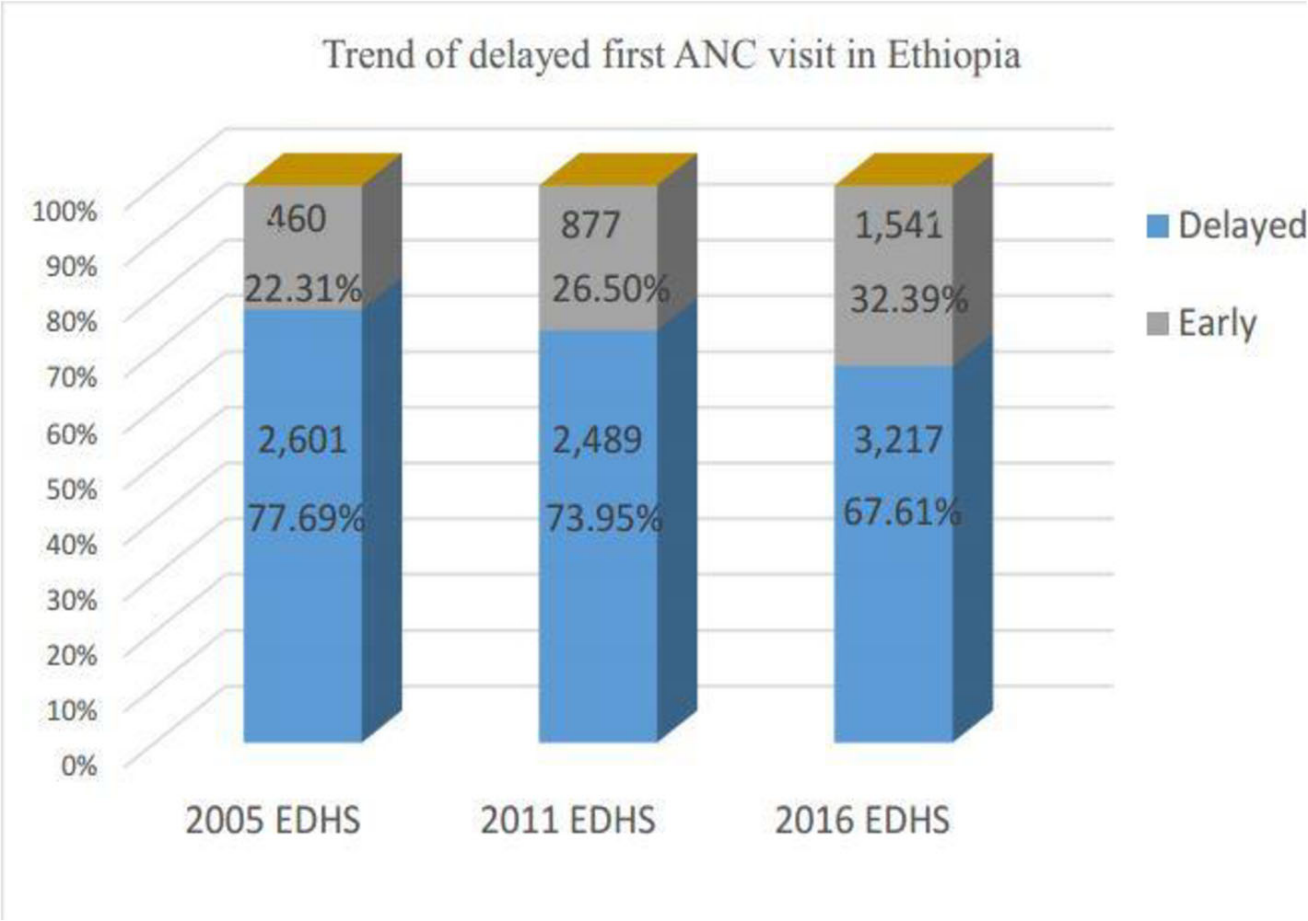
The trend of delayed first ANC visit in Ethiopia on three recent Consecutives EDHS

A total of 10,184 women (2061 in 2005, 3366 in 2011, and 4757 in 2016) were included for this study. Overall, 77.69, 73.95, and 67.61% of women had delayed their first ANC visit in 2005, 2011, and 2016 EDHSs respectively (Fig. 2). From the three consecutive surveys, more than 45% of the mothers were in the age group of 25–34 years and had nearly similar mean ± SD age of 28 ± 6 years. All most (> 85%), of the women, were married in 5 years preceding the survey in three consecutive surveys. Regarding educational status, 37.3, 47, and 46% of women had formal education in each survey year respectively (Table 1).

**Table 1:** Sociodemographic characteristics of respondents

Explanatory variable		EDHS years		
		2005 Frequancy (percentage)	2011 Frequancy (percentage)	2016 Frequancy (percentage)
First ANC vist	Early	460(22.31)	877(26.5)	1541(32.39)
	Delayed	2601(77.69)	2489(73.95)	3217(67.61)
Maternal age (years)	15–24	588(28.51)	947(28.13)	1233(25.90)
	25–34	1002(48.58)	1638(48.61)	2488(52.29)
	35–49	473(22.91)	783(23.26)	10,377(21.81)
	Mean ± SD	28.36 ± 6.8	28.59 ± 6.62	28.7 ± 6.6
Maternal education	No education	1286(62.39)	1780(52.89)	2569(54.01)
	education	776(37.31)	1586(47.11)	2188(45.99)
Marital status	Married	1868(90.62)	2859(84.95)	4419(92.89)
	Not married	194(9.38)	507(15.05)	339(7.11)
Head of houshold	Male	1753(85.06)	2750(81.71)	4066(85.48)
	female	308(14.94)	615(18.29)	691(4.52)
House hold size	1–4	663(32.15)	1092(32.44)	1677(35.24)
	5–22	1398(67.85)	2271(67.49)	3081(64.76)
Media exposure	No	962(46.66)	917(27.23)	2735(57.49)
	Yes	2000(53.34)	2447(72.69)	2023(42.57)
Wealth index	Poor	508(24.65)	1030(30.58)	1727(36.30)
	Middle	408(19.78)	616(18.31)	993(20.87)
	Rich	1146(55.58)	1720(51.11)	2038(42.83)
parity	Primiparous	413(20.04)	753(22.37)	1119(23.52)
	Multiparous	1649(79.96)	2613(77.62)	3638(76.48)
Ever had terminated preginancy	No	1853(89.9)	3007(89.35)	4322(90.68)
	Yes	208 (10.1)	359(10.65)	435(9.14)
Wanted last pregnancy	Un Wanted	388(18.8)	342(10.15)	324(6.81)
	Wanted	1674(81.2)	3024(89.85)	4433(93.19)
Residence	urban	439(21.32)	894(26.54)	870(18.29)
	rural	1621(78.68)	2472(73.46)	3887(81.79)
Distance from health facilities	Big problem	1241(60.22)	2144(63.71)	2397(50.39)
	Not big problem	820(39.78)	1219(36.150	2359(49.81)
Communitymedia usage	Low	696 (33.75)	1867(53.79)	2773(58.29)
	High	1366 (66.25)	1604 (46.21)	1984(41.71)
Community poverty level	Low	245(11.91)	1734 (49.96)	2438(51.24)
	High	1816 (88.09)	1737(50.04)	2320(48.76)

### Multilevel model parameter results

#### Random effect and model comparison

As indicated from Table 2, the ICC in the null model was 19% which indicate the variations of delayed first ANC visit among study subjects were attributed to the difference at the cluster level.

**Table 2:** Parameters and model fit statistics for multilevel regression analysis models

Parametres	Null model (null0	Model2	Model3	Model4
Community level variance (SE)	0.779 (0.104)	0.71(0.096)	0.52(0.077)	0.51(0.081)
ICC	0.192	0.176	0.136	0.134
MOR	2.28	1.96	1.51	1.49
PCV	Reff	8.3%	29.2%	30.2%
Model fittness				
Deviance	6043.85	5904.43	5743.23	5508.62
LR test vs. logistic model	302.88***	114.85 ***	158.62**	195***
Mean VIF	–	1.43	2.78	2.28

* = *P*-value < 0.05. ** = *P* value < 0.01. *** = *P* value < 0.001

*ICC* Inter cluster corrolation cofficent*, MOR* Median odds ratio*, PCV* proportional change in variance

The MOR value (2.28) in the null model, also revealed that the odds of being delayed for first ANC booking among study subjects was different between clusters by this averege median value.

Furthermore, the PCV value in the final model showed that about 30% of the variation in delayed first ANC visit among study subjects was explained by both the individual and community level factors. Deviance was used for model comparison and fit. Model four, which had the lowest deviation, was the best-fitted model (5508.62) (Table 2).

#### Multi-level analysis of factors associated with delayed first ANC visit

All variables (both individual-level and community-level variables) which have *p*-value< 0.20 in the bivariable analysis was eligible for multivariable analysis but ever had a termination of pregnancy has a *p*-value = 0.8, so it was omitted from further analysis. Finally, the adjusted OR with 95% CI and *p*-value < 0.05 in the final model was reported.

Based on the final model result, maternal education wanted pregnancy for the last child, residence, and region were found to be significantly associated with delayed first ANC visit. But, the remaining independent variables had no significant association with the outcome variable. Educated women were 29% less likely to have delayed first ANC visit than women with have no formal education [AOR = 0.71; 95%CI; 0.60, 0.84]. Women who had an unwanted pregnancy for the last child were 1.41 times more likely to have delayed the first ANC visit as compared to those who had wanted pregnancy [AOR = 1.41;95%CI; 1.04, 1.89]. The odds of having delayed first ANC visit among women of rural residence was 1.68 times more as compared to women of urban [AOR = 1.68;95%CI; 1.19, 2.38]. The odds of having delayed first ANC visit among women who live in SNNRP, Benshangul Gumuz, and Oromia was 3.38 [AOR = 3.38; 95%CI: 2.01, 5.67], 3.09 [AOR = 3.09; 95%CI: 1.34, 7.15] and 2.33 [AOR = 2.33; 95%CI: 1.41, 3.86] times higher as compared to Addis Ababa respectively (Table 3).

**Table 3:** A multilevel analysis of factors associated with delayed first ANC visit among reproductive age women in Ethiopia, data from 2016 EDHS

Explanatory variable		Model – 2	Model – 3	Model- 4
		AOR, [95% CI]	AOR, [95% CI]	AOR, [95% CI]
Age category	15–24	1.00	–	1.00
	25–34	0.71[0.57, 0.85]*	–	0.86[0.63,1.01]
	35–49	0.70[0.55, 0.91]	–	0.79[0.62,1.02]
Maternal education	Not educated	1.00	–	1.00
	Educated	0.68[0.57, 0.81]**	–	0.71[0.60,0.84]***
Marital status	merried	1.00	–	1.00
	Not merried	0.92[0.68, 1.22]	–	0.98[0.73,1.31]
Head of houshold	male	1.00	–	1.00
	female	0.78[0.62, 0.96]*	–	0.81[0.65,1.01]
Parity	Prim-pareous		–	1.00
	Multi- pareous	1.24[1.00, 1.50]*	–	1.18[0.96,1.44]
Wanted pregnancy	wanted		–	1.00
	unwanted	1.43[1.06, 1.93]*	–	1.41[1.04,1.89]*
Wealth index	poor	1.16[0.95,1.44]	–	0.97[0.78,1.21]
	middle	1.06[0.87,1.31]	–	0.89[0.72, 1.11]
	rich	1.00	–	1.00
Media exposure	No	1.00	–	1.00
	Yes	**0.81[0.68, 0.95**]*	–	0.88[0.75,1.05]
Household size	1–4	1.00	–	1.00
	5–19	0.12[0.94,1.32]	–	1.06[0.90,1.25]
Distance from health facilities	Big problem	1.00	–	1.00
	Not Big problem	1.01[0.85, 1.18]	–	1.15[0.97,1.36]
Community level factors				
Residence	urban	**–**	1.00	1.00
	rural	–	1.94 [1.41, 2.67]^***^	1.68 [1.19, 2.38]*
Region	Tigray	–	1.79[1.07, 3.01]*	1.67[0.99,2.83]
	Afar	–	1.89[0.78,4.56]	1.67[0.68, 4.11]
	Amhara	–	1.49 [0.91,2.44]	1.26 [0.77, 2.11]
	Oromia	–	2.46 [1.50, 4.04]***	2.33 [1.41,3.86]*
	Somalia	–	2.29 [1.20, 4.39]*	1.92 [0.99, 3.73
	B/gumiz	–	3.26 [1.43, 7.46]*	3.09 [1.34, 7.15]*
	SNNPR	–	3.58 [2.15, 5.93]***	3.38 [2.01, 5.67]***
	Gambella	–	1.49 [0.46, 4.89]	1.47 [0.44, 4.84]
	Harare	–	0.81 [0.23, 2.82]	0.71 [0.19, 2.52]
	Dire dewa	–	0.49 [0.19,1.29]	0.46 [0.17, 1.23]
	Addis ababa	1.00	**1.00**	1.00
Com.poverty	low	1.00	1.00	1.00
	high	1.00	1.19[0.77,1.85]	1.21[0.77,1.89]
Com. Media	low	1.00	1.00	1.00
	high	1.00	0.99 [0.64, 1.52]	0.92 [0.59, 1.45]

* = *P*-value < 0.05, ** = *P* value < 0.01, *** = *P* value < 0.001

*AOR* adjusted odds ratio*; Com. Media* community media usage

*CI* confidence interval*; Com. Poverty* community poverty status

#### Spatial and incremental autocorrelation analysis of delayed first ANC visit among reproductive-age women in Ethiopia, 2005, 20,011, and 2016 EDHS

**Fig. 3:**
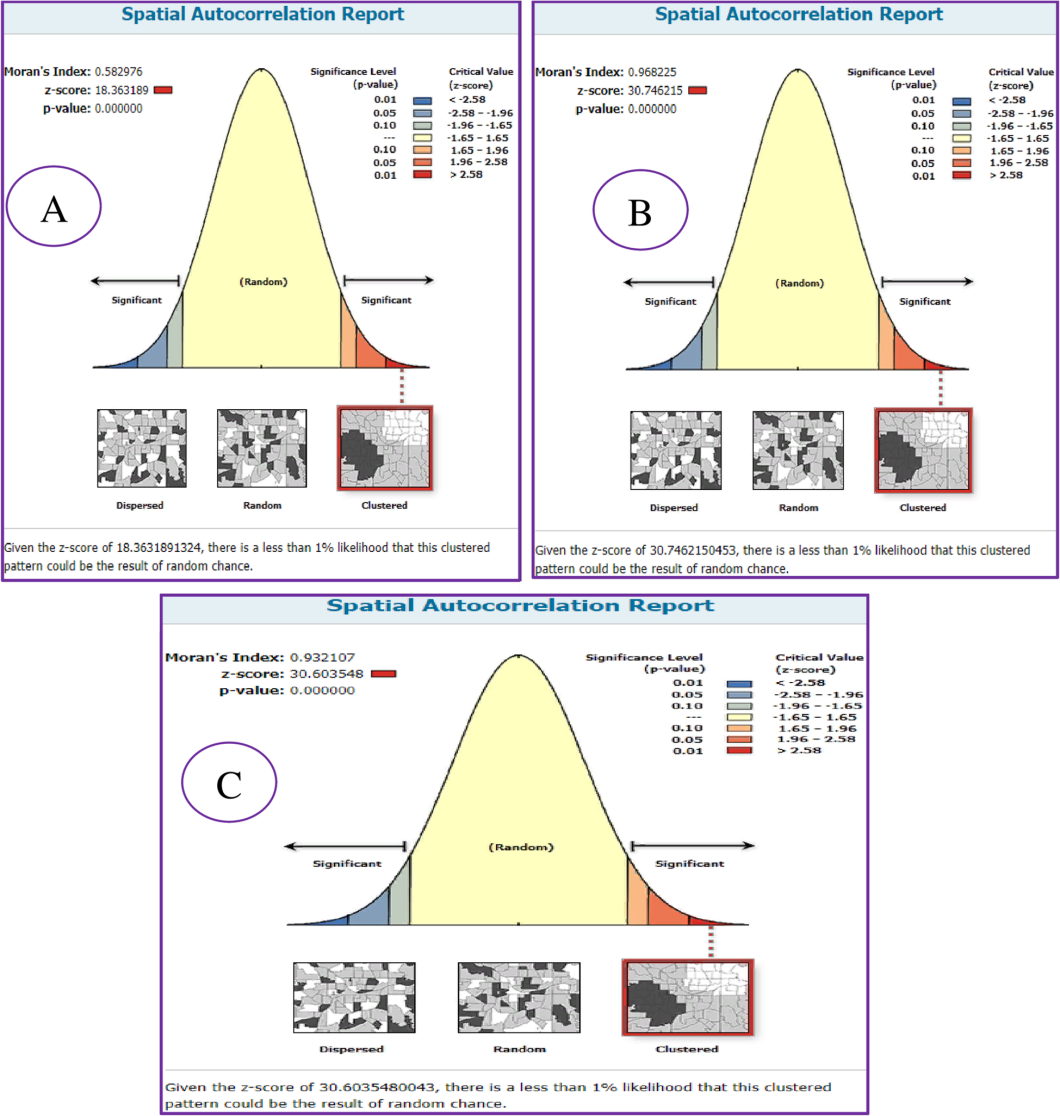
Spatial autocorrelation of delayed first ANC visit in Ethiopia EDHS 2005 (**A**), EDHS 2011(**B**), and EDHS 2016(**C**) plotted using arcMap 10.7

Spatial distribution of delayed first ANC visit among reproductive-age women showed significant spatial variation across the country over time. In 2005, 20,011, and 2016 EDHS, the spatial distribution of delayed first ANC visit among reproductive-age women were found to be non-random (clustered) with Global Moran’s I value 0.58, 0.96, and 0.93 with (*p* < 0.0001) respectively (Fig. 3).

The incremental autocorrelation result showed that statistically significant z-scores indicated at one peak distance in both 2005 EDHS at 381.602Km; 8.60(distances; Z-score) and 2011 EDHS at 160.23 Km; 26.4 (distances; Z-score). Whereas it has two peak distances 151.13 km; 27.73 (distances; Z-score) and 180.46Km; 28.19 (distances; Z-score) in 2016 EDHS in where spatial processes promoting clustering are most pronounced detected by 10 distance bands.

#### Spatial distribution and interpolation of delayed first ANC visit in Ethiopia

**Fig. 4:**
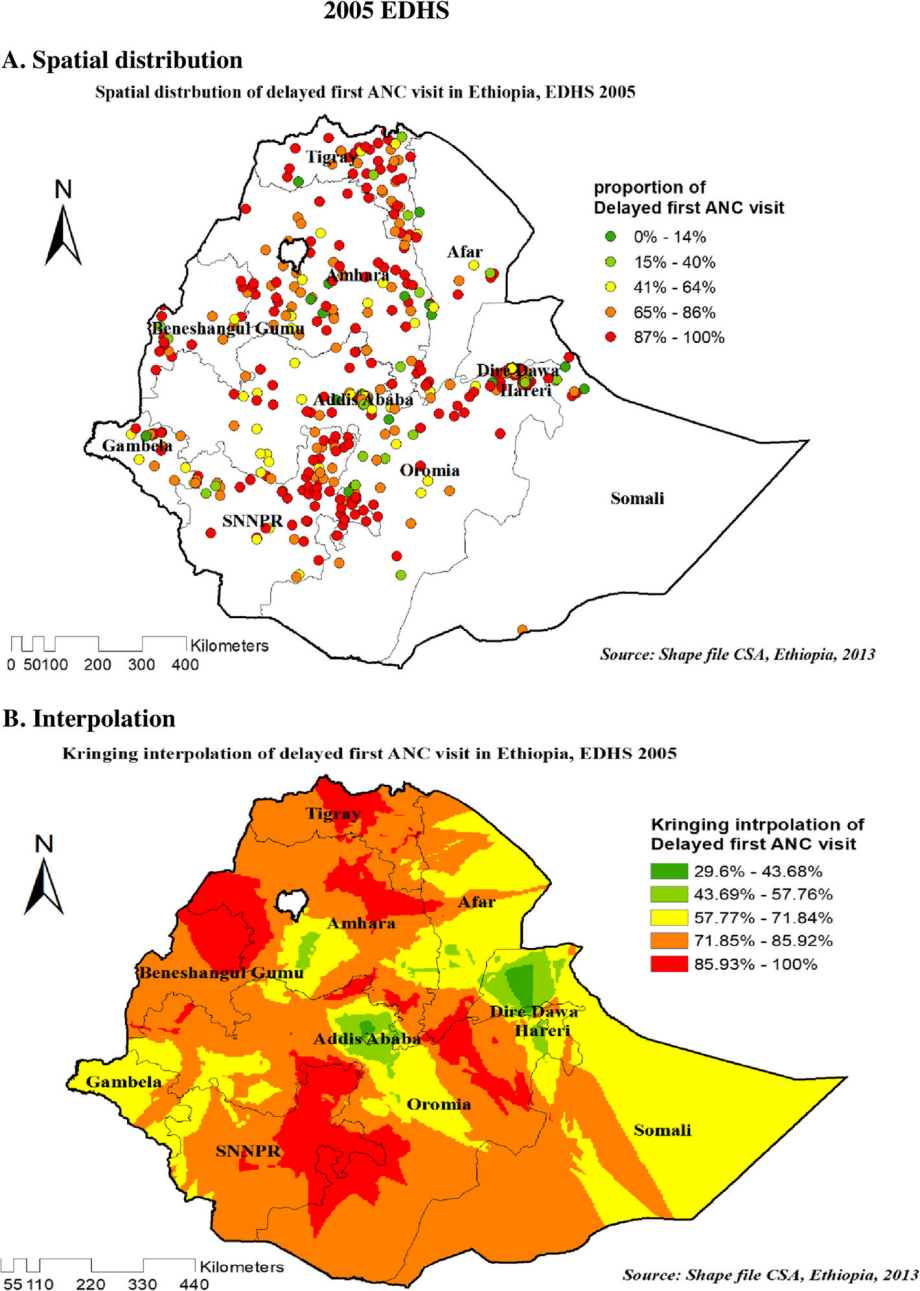
Spatial distribution (4**A**) and interpolation (4**B**) of delayed first ANC visit in Ethiopia EDHS 2005 plotted using arcMap 10.7

**Fig. 5:**
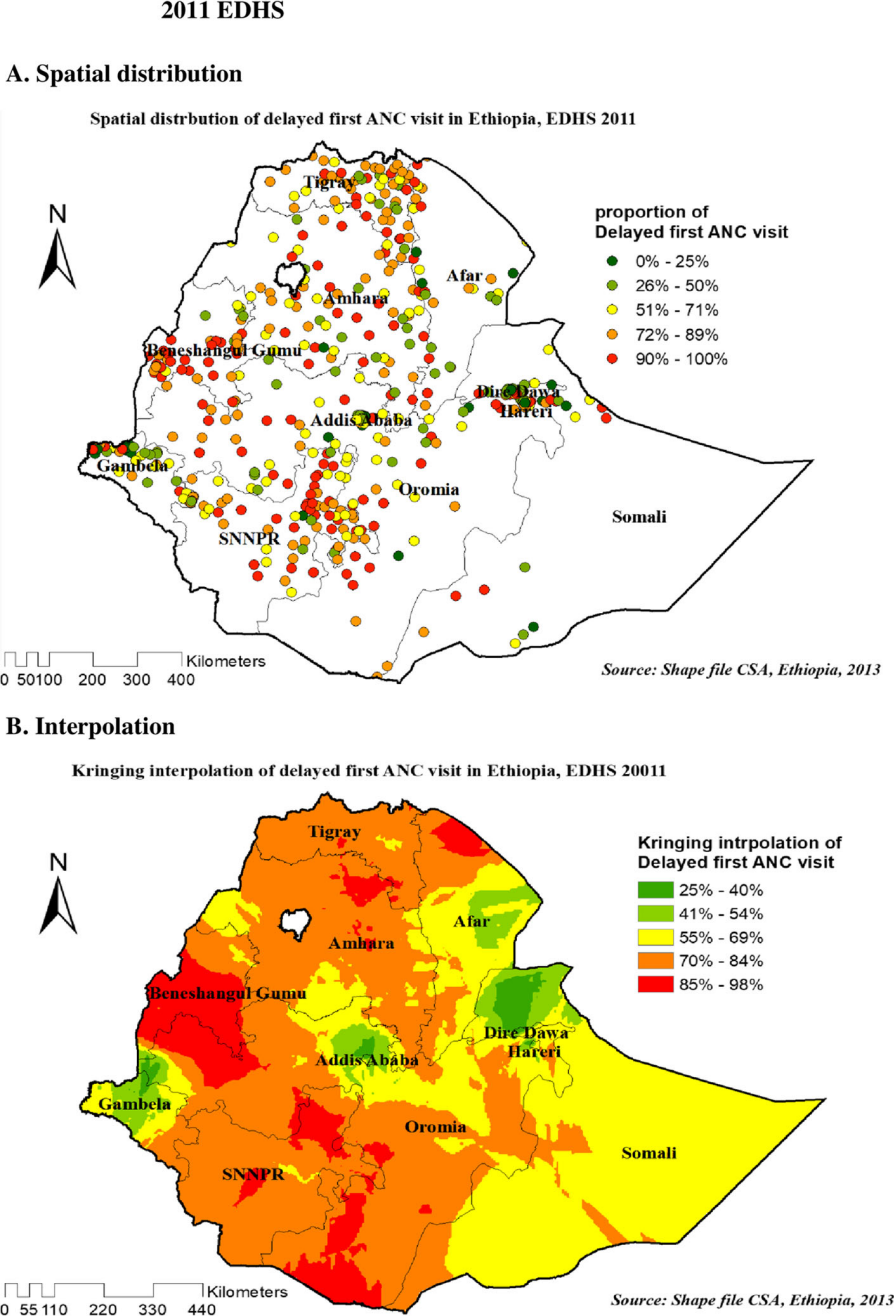
Spatial distribution (5**A**) and interpolation (5**B**) of delayed first ANC visit in Ethiopia EDHS 2011 plotted using arcMap 10.7

**Fig. 6:**
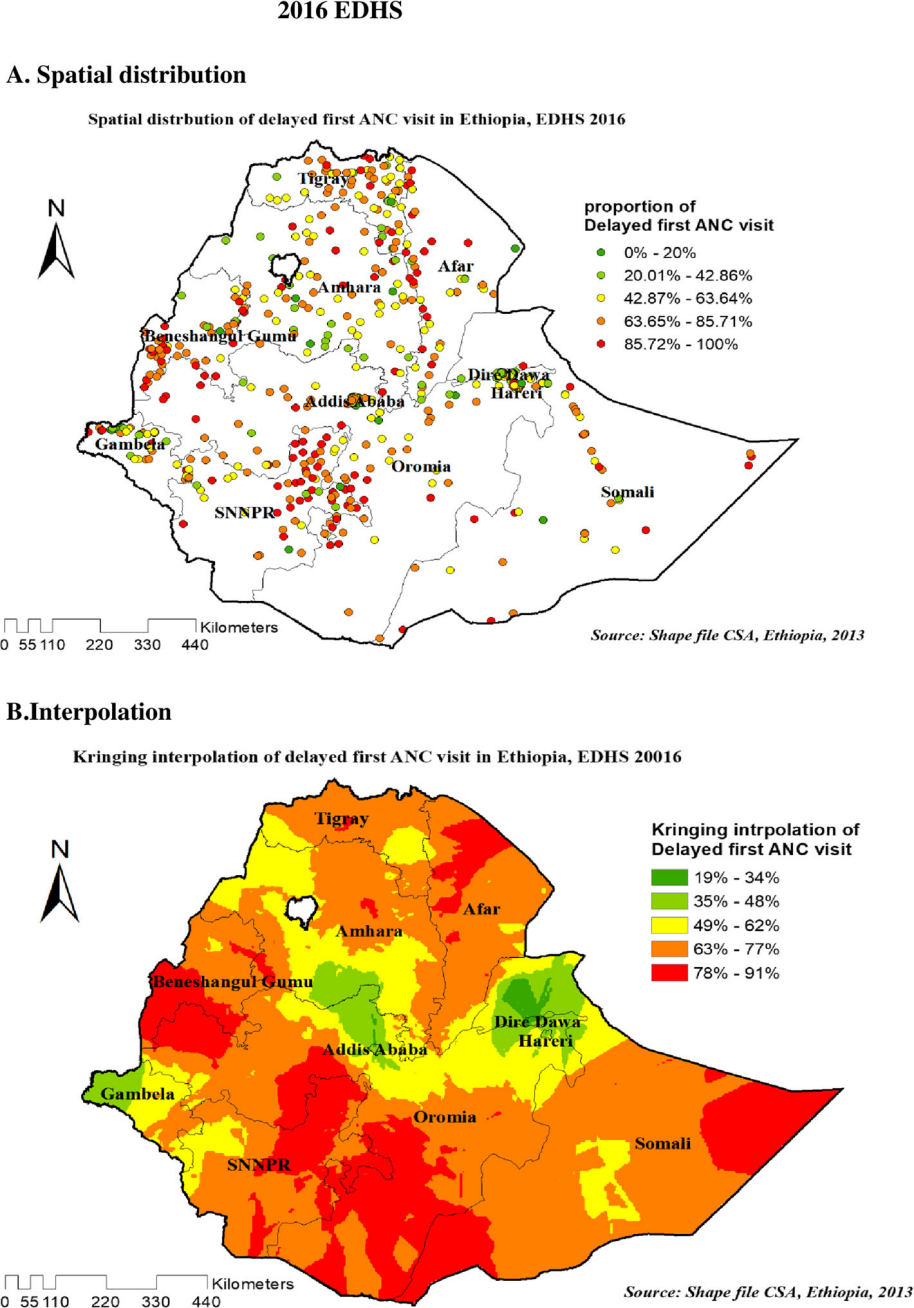
Spatial distribution (6**A**) and interpolation (6**B**) of delayed first ANC visit in Ethiopia EDHS 2016 plotted using arcMap 10.7

As shown in the following figures, The red dots represent a more concentrated clustering of delayed first ANC visits among reproductive-age women, whereas the green dots suggest a smaller proportion of delayed first ANC visits among reproductive-age women. Figure 4A, Fig. 5A and Fig. 6A showed that spatial variation was found in delayed first ANC visit at regional levels in each EDHS (Fig. 4A, Fig. 5A, and Fig. 6A).

Figures 4B, Fig. 5B, and Fig. 6B showed Kriging interpolation methods of predicting delayed first ANC visit among reproductive-age women in each consecutive EDHS over the area which were increases from green to red-colored areas. The red color denotes high-risk areas, while the green color denotes low-risk areas for reproductive-age women’s expected having delayed first ANC visit. The prevalence of high-risk areas predicted delayed first ANC visit was extremely high and ranges from 85 to 100% in all EDHS.

Figure 4B on 2005 EDHS revealed that eastern and western part of Amhara, Northeast B/Gumuz, eastern SNNP (south nation nationalities and peoples of Ethiopia), and eastern Oromia regions have predicted more delayed first ANC visit compared to other regions. In contrast, the predicted lower delayed first ANC visit was found in Addis Ababa, Dire Dawa, Harari, and around the center of Amhara (Fig. 4B). Based on EDHS 2011, Kriging interpolation predict that the highest delayed first ANC visit was detected in the Northern part of Amhara, Northeast Afar, south B/Gumze, wast and sowth Oromia regions and central SNNP whereas, predicted relatively lower delayed first ANC visit located in the Addis Ababa, Gambella, North Somali, and Eastern Afar regions (Fig. 5B). In the recent EDHS, EDHS 2016 data Kriging interpolation predicted that border of B/Gumz and Oromia, North SNNP, South Oromia, Northern Afar, and the horn of Somali region predicted the highest prevalence of unintended pregnancy, whereas southern Amhara, Addis Ababa, North Somali, Diredewa and Western Gambella regions predicted relatively low unintended pregnancy (Fig. 6B).

#### Hot spot analysis (Getis-Ord Gi* statistic) of the three surveys

**Fig. 7:**
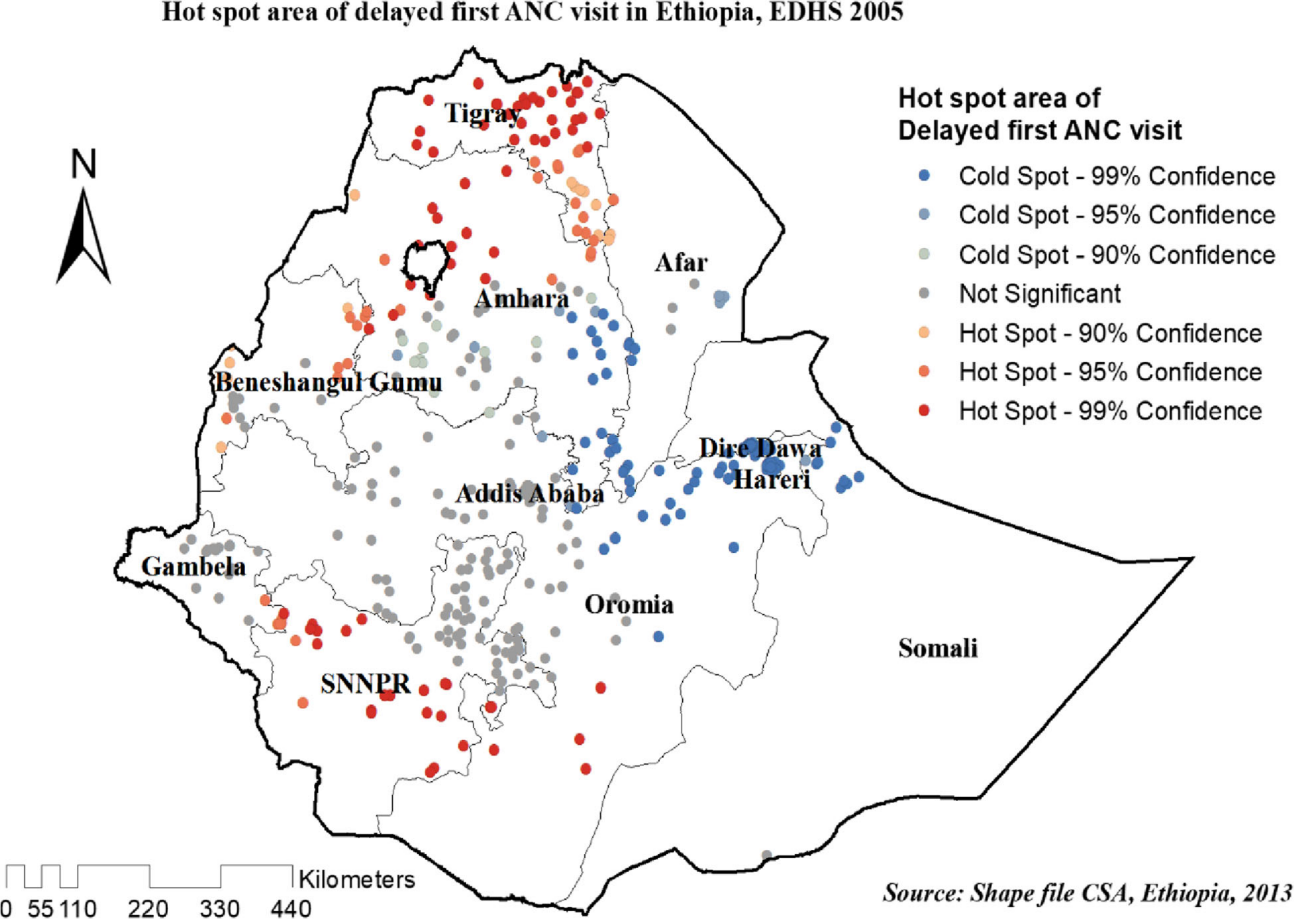
Hot spot areas of delayed ANC first visit in Ethiopia, EDHS 2005 plotted using arcMap 10.7

The spatial distribution of delayed first ANC visit was almost similar in the three survey years. In EDHS 2005 hot spot areas of delayed first ANC visit were detected in entire of Tigray, northern west Amhara, and the southern part SNNRP region, Whereas, eastern Amhara, DireDawa, Harari, and Addis Ababa regions of Ethiopia were less risk area (Fig. 7).

**Fig. 8:**
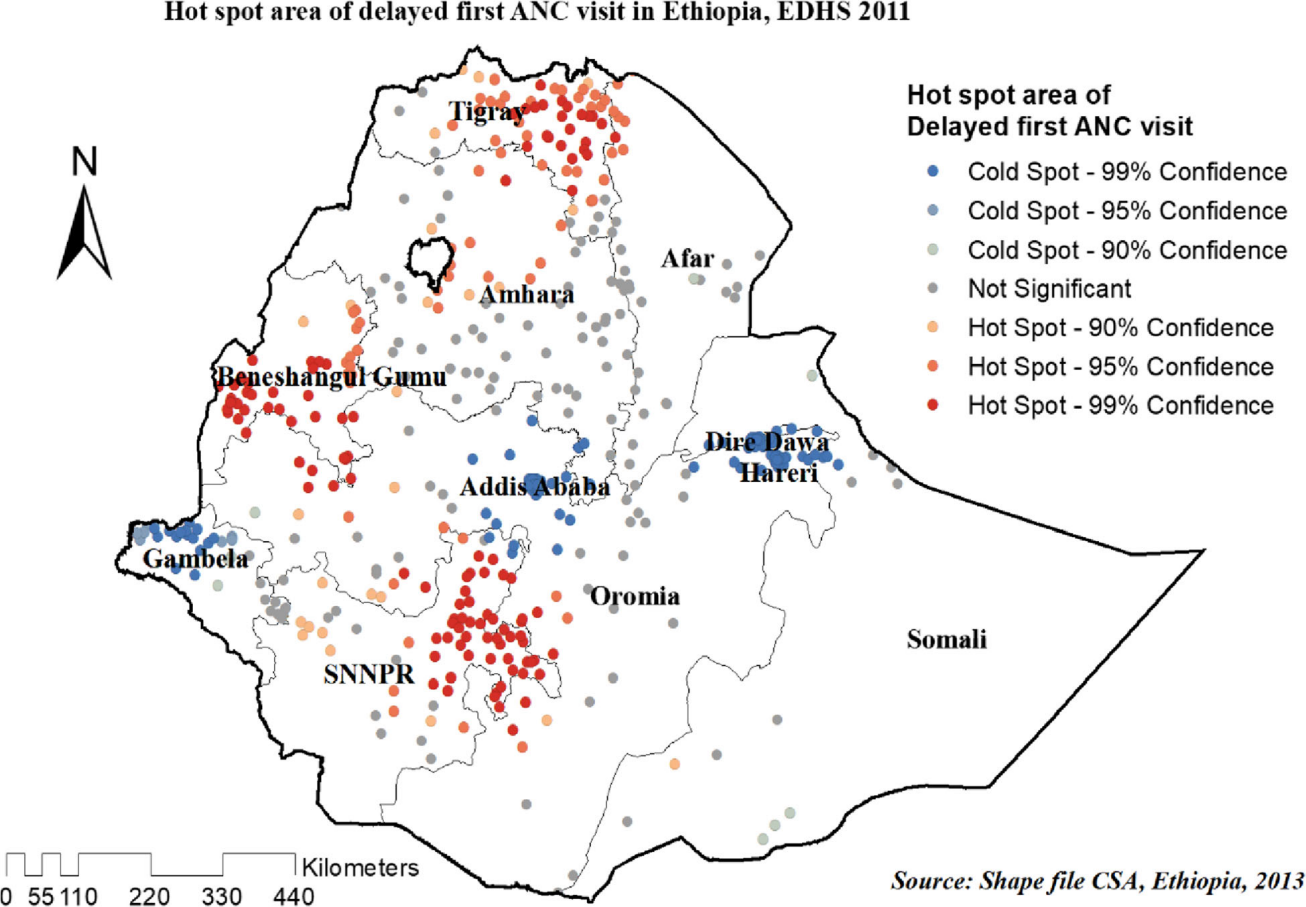
Hot spot areas of delayed ANC first visit in Ethiopia, EDHS 2011 plotted using arcMap 10.7

**Fig. 9:**
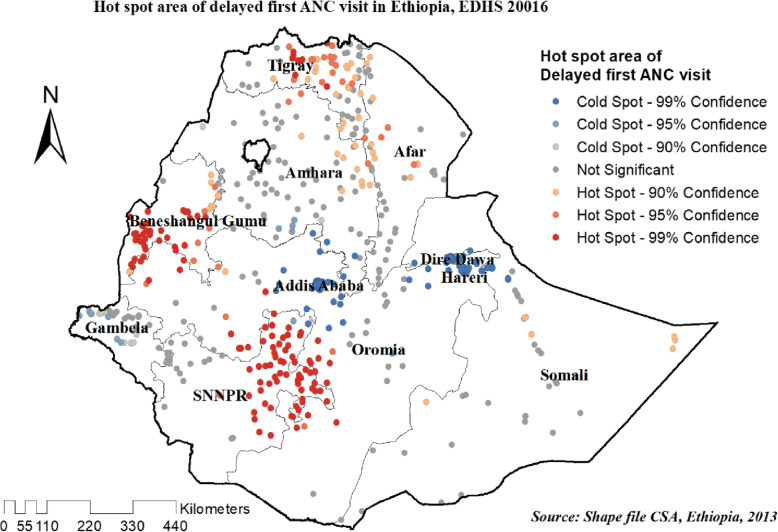
Hot spot areas of delayed ANC first visit in Ethiopia, EDHS 2016 plotted using arcMap 10.7

In 2011, and 2016 EDHS high clustering of delayed first ANC visit detected in most parts of Tigray, the western part of Benshangul Gumuz, southern Oromia, and the northern part of the SNNPR region Whereas, cold spot area was detected in DireDawa, Harari, Addis Ababa and Gambella regions of Ethiopia (Figs. 8 and 9).

#### Spatial scan statistics analysis of three survey years

**Fig. 10:**
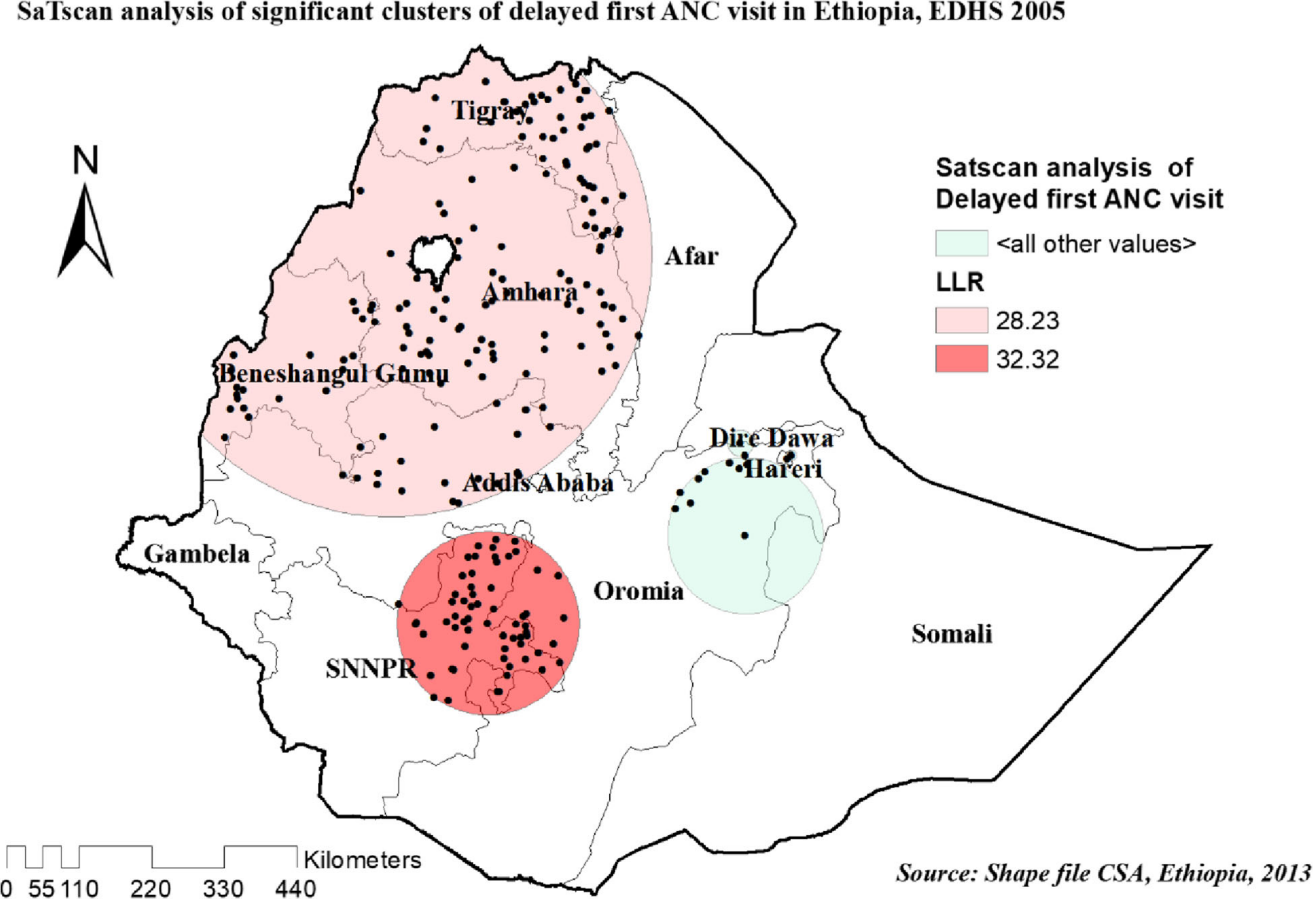
Significant clusters of delayed first ANC visit spatial window in Ethiopia, EDHS 2005 plotted using arcMap 10.7

Most likely primary and secondary clusters of delayed ANC first visit among women of the reproductive age group were identified. In 2005 EDHS, among a total of 122 most likely clusters, 62 of them were primary clusters. These were located between the border of Oromia and Eastern SNNPR centered at 7.065937 N, 38.082394 E, with a 137.79 km radius. Women who were living in the SaTScan window were 30% more likely to have delayed their first ANC visit (RR = 1.30, LLR = 32.31, *P*-value< 0.001) (Table 4, and Fig. 10).

**Table 4:** SaTScan analysis result of unintended pregnancy among reproductive age women in Ethiopia, 2005,2011 and 2016 EDHS

EDHS	EDHS 2005		EDHS 2011		EDHS 2016	
Cluster	Primery	Secondary	Primery	Secondary	Primery	Secondary
Number of clusters	1 [62]	2 [160]	1 [145]	2 [41]	1 [198]	2 [31]
Coordinate /Radius	7.065937 N, 38.082394 E /137.79 km	12.045916 N, 36.721581 E /393.93 km	12.706300 N, 36.064266 E /485.52 km	4.709865 N, 38.656818 E /320.45 km	7.059671 N, 35.488608 E /412.55 km	4.123765 N, 38.589911E /89.50 km
Population	309	711	1077	304	1588	316
Cases	275	579	793	231	973.5	193.74
RR	1.30	1.23	1.30	1.23	1.35	1.25
LRR	32.31	28.23	40.79	12.36	83.21	14.89
*P*-value	*P* = 0.001	*P* = 0.001	*P* = 0.001	*P* = 0.004	*P* = 0.001	*P* = 0.001

**Fig. 11:**
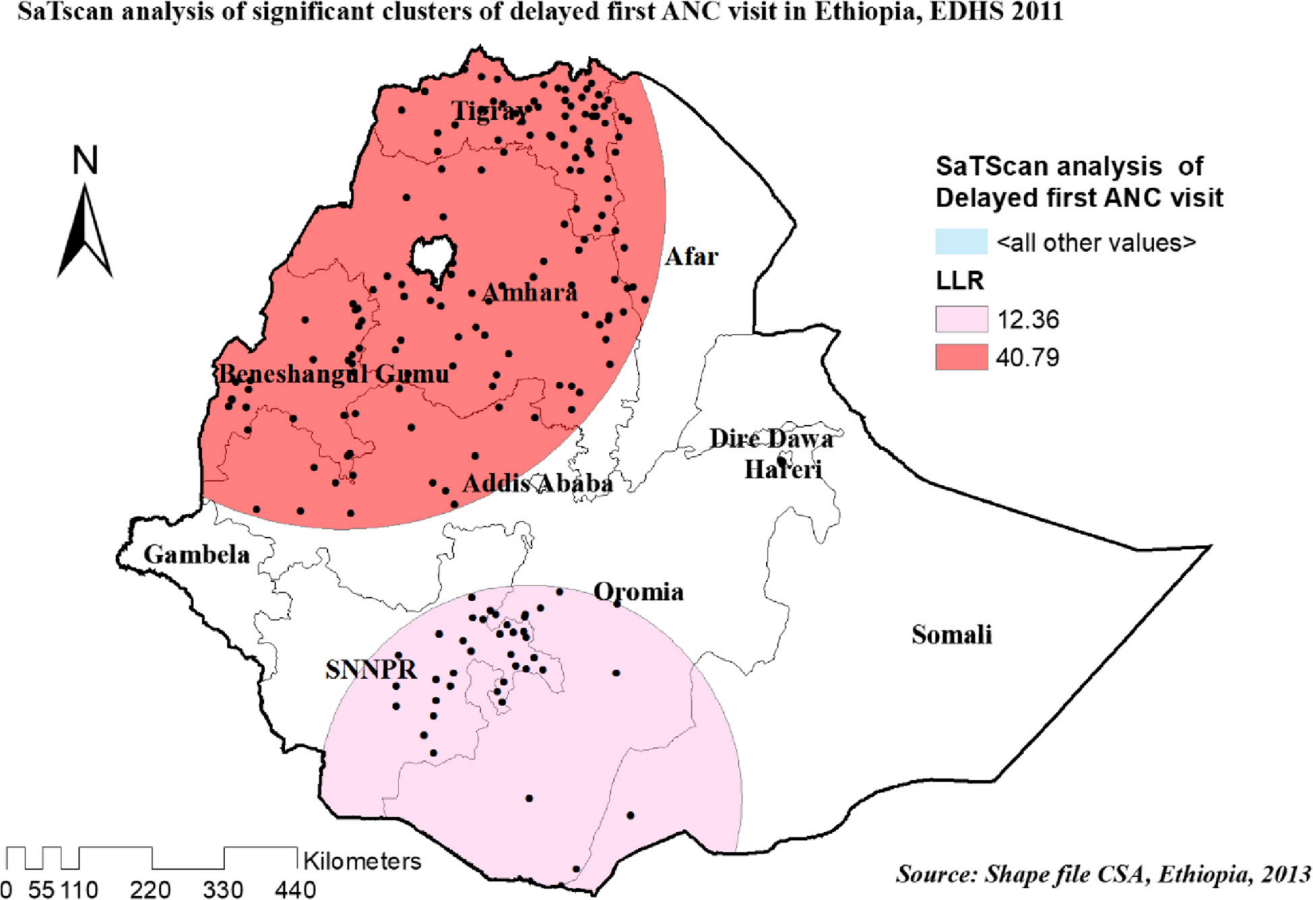
Significant clusters of delayed first ANC visit spatial window in Ethiopia, EDHS 2011 plotted using arcMap 10.7

In 20,011 EDHS, among a total of 186 most significant clusters, 145 of them were primary clusters. These were located in the entire Tigray, B/Gumuz, Amhara western part of afar and northwest Oromia regions centered at 12.706300 N, 36.064266 E with 485.52 km radius. Similarly, with the 2005 EDHS, women who were living in the SaTScan window were 30% more likely to have delayed the first ANC visit (RR = 1.30, LLR = 40.79, *P*-value< 0.001) (Table 4, and Fig. 11).

**Fig. 12:**
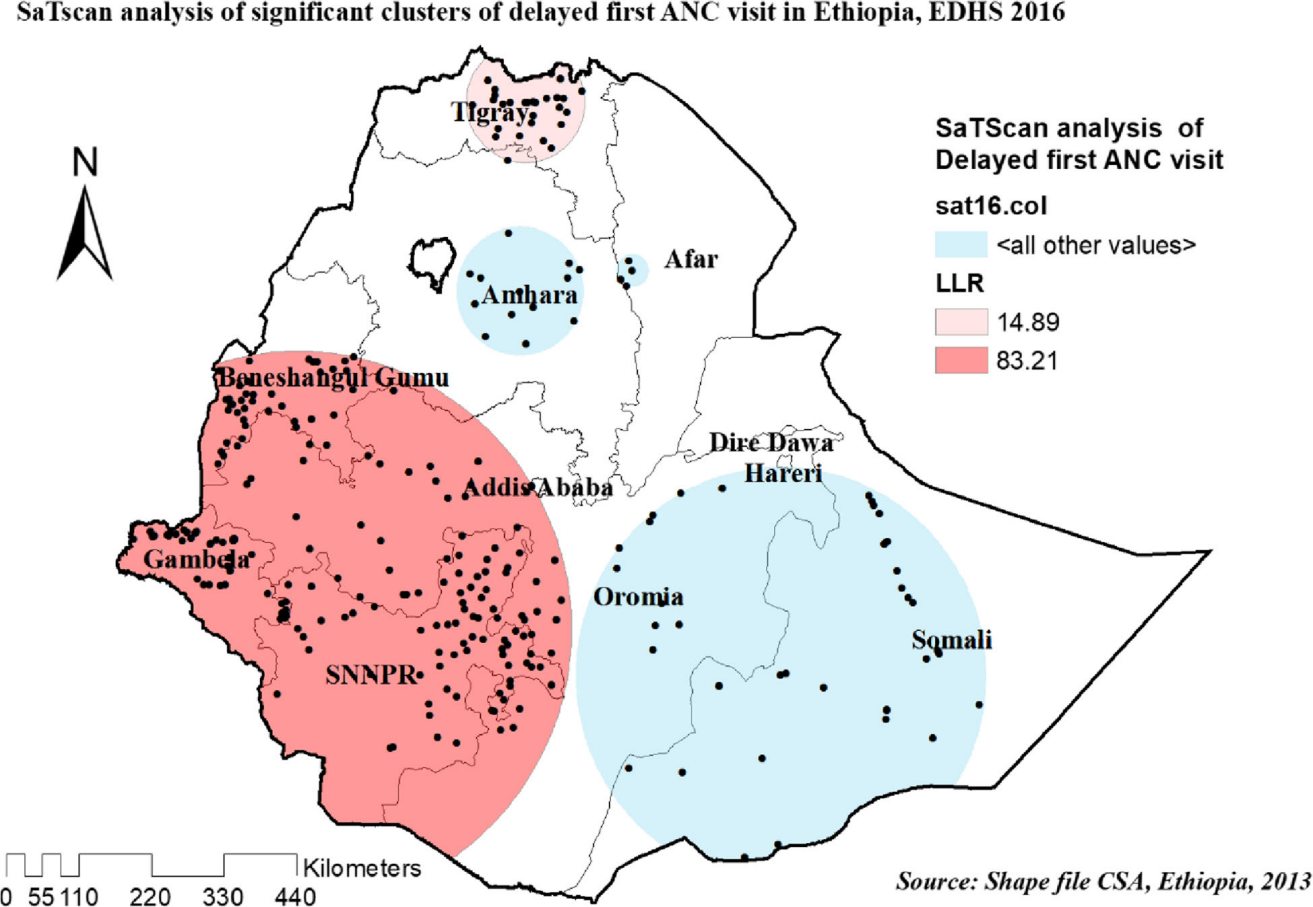
Significant clusters of delayed first ANC visit spatial window in Ethiopia, EDHS 2016 plotted using arcMap 10.7

In recent EDHS 2016, the spatial scan statistics identified a total of 229 significant clusters identified, among these 198 were primary clusters which were located in the entire SNNPR, Gambella, northern B/Gumuz, and western Oromia regions, centered at 7.059671 N, 35.488608 E with 412.55 km radius. Women who were living in the SaTScan window were 35% more likely to have delayed the first ANC visit (RR = 1.35, LLR = 83.21, *P*-value< 0.001) (Table 4, and Fig. 12).

## Discussion

This study shows that 67.6% (95%CI: 66.3 to 68.9%) of women had delayed to take their first ANC visit in recent 2016 EDHS. This finding was almost similar to a systematic review and meta-analysis study conducted in Ethiopia (64%) [17]. However, the research is done in Bangladesh revealed a small prevalence of delayed first ANC visit (13.5) [20]. This discrepancy is may be due to differences in health service coverge and health-seeking behavior of the societies in western countries.

According to our finding factors associated with delayed first ANC visit were women with no education, women with an unwanted pregnancy, and residence of women. In our study, as compared with educated women, uneducated women have more likely to have delayed first ANC visit. This finding was supported by a systematic review and meta-analysis study conducted in Ethiopia [17], research done in Debre Markos, Ethiopia [10], and research done on Tribal women of Bangladesh [20]. This shows that increasing women’s educational status has a positive impact on timely booking of their first ANC visit.

In this study women who had an unwanted pregnancy for their last child was associated with an increased occurrence of delayed first ANC visit. This is in line with a systematic review and meta-analysis study conducted in Ethiopia [17, 33], research done in Tanzania [34], a study in Rwanda [25]. This might due to lack of information, lack of decision-making authority, lack of resources, or cultural factors, women with unintended pregnancy may delay ANC initiation [33]. Unintended pregnancies are often linked to social and cultural determinants of health-seeking behaviors, such as sexual harassment, inadequate use of family planning, which are barriers to health care access, which leading to the late initiation of ANC [33].

Living in a rural area was found to be associated with an increased occurrence of delayed first ANC visit in women. This is in line with studies conducted in Ethiopia [17, 23]. This might due to that the the distance from the healthcare facility and the quality of ANC services provided may be mediating such outcomes. Other factors like knowledge about the importance of timing and frequency of ANC visits also have a contribution [23]. The spatial distribution of delayed first ANC visit was almost similar in the three survey years. In EDHS 2005 hot spot areas of delayed first ANC visit were detected in entire of Tigray, Northern west Amhara, and the southern part SNNRP regions, whereas in 2011, and 2016 EDHS high clustering of delayed first ANC visit was detected in most parts of Tigray, the western part of Benshangul Gumuz, southern Oromia and the northern part of SNNPR regions. It was supported by multivariate analysis in this study which shows that women who live in SNNRP, Benshangul Gumuz, and Oromia regions had higher odds of delayed first ANC visit as compared to Addis Ababa. The inclusion of nationally representative datasets gave this study its merits, allowing it to be generalised to all reproductive-age women in the study context. Secondly, the estimates of the study were done after the data were weighted to make it representative at the national and regional levels. This study included the spatiotemporal distribution of delayed first ANC visit which was spatially autocorrelated. However, this study is not free from limitations, Since 21 clusters did not have coordinated data we excluded it in the analysis which may affect the estimated result. The data used in this study are cross-sectional data, which limit the conclusions about the causality of factors in the dependent variable. Since it was secondary data some important variables like behavioral factors and service utilization-related parameters were missed.

## Conclusion

According to this study, even if the proportion of pregnant women who had delayed the first ANC visit decreased in each consecutive survey, the magnitude of the problem is still high. Maternal education was positively associated with delayed first ANC visit, whereas having unwanted pregnancy for the last child and rural residence have negatively associated with delayed first ANC visit. In Ethiopia delayed first ANC visit varies in each EDHS period and across regions. In recent two EDHS, high clustering of delayed first ANC visit was detected in most parts of Tigray, the western part of Benshangul Gumuz, Southern Oromia, and the northern part of SNNPR regions.

Therefore, The governmental and non-governmental organizations should work by giving special attention to those groups of women who had a higher prevalence of delayed ANC visit such as unwanted pregnancy for the last child and living in rural residences is needed. Women’s education should be encouraged and better expand. A targeted intervention program is also required in highly affected regions of Ethiopia. A further case-control study will be needed to dig out the proximal factors contributing to the delayed first ANC visit.

## Data Availability

Data is available online and you can access it from www.measuredhs.com.

## Abbreviations

AIC: Kkakies information criteria
ANC: Anti natal care
AOR: Adjusted odds ratio
CI: Confidence interval
COR: Crude odds ratio
CSA: Central statistical agency
DHS: Demographic and health survey
EDHS: Ethiopian demographic and health survey
GWR: Geographically weighted regression
ICC: Intraclass correlation coefficient
ICF: International community fund
KR: Kids record
LLR: Log-likelihood ratio
LR: Likelihood ratio
MOR: Median odds ratio
PCV: Proportion of change in variance
SNNPR: South nation nationalities and peoples of the region
VA: Variance of the area level
VIF: Variance inflation factors

## References

[1] Organization, W.H (2015). Strategies towards ending preventable maternal mortality (EPMM).

[2] Orginization, W.h. WHO.MaternalHealth |WHO| RegionalOfficeforAfrica [Internet]..p.1–13. . 2018; Available from: Availablefrom: https://afro.who.int/health-topics/maternal-health.

[3] Organization, W.h., WHO recommendation on antenatal care contact schedules. 28 March 2018.

[4] Teshale AB, Tesema GA (2020). Prevalence and associated factors of delayed first antenatal care booking among reproductive age women in Ethiopia; a multilevel analysis of EDHS 2016 data. PLoS One.

[5] Okedo-Alex IN, Akamike IC, Ezeanosike OB, Uneke CJ (2019). Determinants of antenatal care utilisation in sub-Saharan Africa: a systematic review. BMJ Open.

[6] Alliance, W.R., Respectful maternity care: the universal rights of childbearing women. Washington, DC. 2011. 2017.

[7] Lincetto O, et al. Opportunities for African Newborns: World Health Organization; 2012. p. 51–62. Available at: https://www.who.int/pmnch/media/publications/aonintro.

[8] Organization, W.H. Trends in maternal mortality: 1990-2015: estimates from WHO, UNICEF, UNFPA, World Bank Group and the United Nations Population Division: executive summary: World Health Organization; 2015. Available at: https://apps.who.int/iris/bitstream/handle/10665/193994/WHO_RHR_15.23_ara.pdf.

[9] Prevention, O.o.D. and H. Promotion, US Department of Health and Human Services: Healthy People 2010. http://www/health/gov/healthypeople/, 2000.

[10] Ewunetie AA, Munea AM, Meselu BT, Simeneh MM, Meteku BT (2018). DELAY on first antenatal care visit and its associated factors among pregnant women in public health facilities of Debre Markos town, north West Ethiopia. BMC Pregnancy Childbirth.

[11] Lincetto, O., et al., Antenatal care. Opportunities for Africa's newborns: Practical data, policy and programmatic support for newborn care in Africa, 2006: p. 55–62.

[12] Unicef, children, food and nutrition and Growing well in a changing world. 2019.

[13] Organization, W.H., WHO recommendation on antenatal care contact schedules. 2018 [cited 2020 7 April].

[14] Nieburg P (2012). Improving maternal mortality and other aspects of women’s health.

[15] Lawn JE, Cousens S, Bhutta ZA, Darmstadt GL, Martines J, Paul V, Knippenberg R, Fogstadt H, Shetty P, Horton R (2004). Why are 4 million newborn babies dying each year?. Lancet.

[16] Victora CG (2010). Socio-economic and ethnic group inequities in antenatal care quality in the public and private sector in Brazil. Health Policy Plan.

[17] Tesfaye G, Loxton D, Chojenta C, Semahegn A, Smith R (2017). Delayed initiation of antenatal care and associated factors in Ethiopia: a systematic review and meta-analysis. Reprod Health.

[18] Centeral intellegence agency (CIA), U.S.A., The world fact book: maternal mortality rate. 2020.

[19] Organization, W.H. WHO antenatal care randomized trial: manual for the implementation of the new model: World Health Organization; 2002. Available at: https://apps.who.int/iris/bitstream/handle/10665/42513/WHO_RHR_01.30.pdf

[20] Karim AR (2019). Assessing barriers for delayed antenatal care services among tribal women of Bangladesh. Int J Public Health Res.

[21] Organization, W.H. Trends in maternal mortality: 1990–2015: estimates from WHO, UNICEF, UNFPA, World Bank Group and the United Nations Population Division: World Health Organization; 2015. Available at: https://apps.who.int/iris/bitstream/handle/10665/194254/9789241565141.

[22] Wolde HF, Tsegaye AT, Sisay MM (2019). Late initiation of antenatal care and associated factors among pregnant women in Addis Zemen primary hospital, South Gondar, Ethiopia. Reprod Health.

[23] Yaya S, et al. Timing and adequate attendance of antenatal care visits among women in Ethiopia. PLoS One. 2017;12(9):e0184934. 10.1371/journal.pone.0184934. 10.1371/journal.pone.0184934 PMC5602662 28922383

[24] Gebresilassie B, Belete T, Tilahun W, Berhane B, Gebresilassie S (2019). Timing of first antenatal care attendance and associated factors among pregnant women in public health institutions of Axum town, Tigray, Ethiopia, 2017: a mixed design study. BMC Pregnancy Childbirth.

[25] Manzi A, Munyaneza F, Mujawase F, Banamwana L, Sayinzoga F, Thomson DR, Ntaganira J, Hedt-Gauthier BL (2014). Assessing predictors of delayed antenatal care visits in Rwanda: a secondary analysis of Rwanda demographic and health survey 2010. BMC Pregnancy Childbirth.

[26] Central Statistical Agency (CSA) [Ethiopia] and ICF (2016). Ethiopia Demographic and Health Survey 2016.

[27] Organization, W.H., WHO antenatal care randomized trial: manual for the implementation of the new model. . Geneva, Switzerland; 2002. 2002.

[28] Liyew AM, Teshale AB (2020). Individual and community level factors associated with anemia among lactating mothers in Ethiopia using data from Ethiopian demographic and health survey, 2016; a multilevel analysis. BMC Public Health.

[29] Merlo J, Chaix B, Yang M, Lynch J, Råstam L (2005). A brief conceptual tutorial of multilevel analysis in social epidemiology: linking the statistical concept of clustering to the idea of contextual phenomenon. J Epidemiol Community Health.

[30] Merlo J, Chaix B, Yang M, Lynch J, Råstam L (2005). A brief conceptual tutorial on multilevel analysis in social epidemiology: interpreting neighbourhood differences and the effect of neighbourhood characteristics on individual health. J Epidemiol Community Health.

[31] McMillen DP. Geographically weighted regression: the analysis of spatially varying relationships: Oxford University Press; 2004. Available at: https://www.jstor.org/stable/30139578.

[32] Kulldorff M (1997). A spatial scan statistic. Commun Stat Theory Methods.

[33] Tolossa T (2020). Association between pregnancy intention and late initiation of antenatal care among pregnant women in Ethiopia: a systematic review and meta-analysis. Syst Rev.

[34] Exavery A, Kanté AM, Hingora A, Mbaruku G, Pemba S, Phillips JF (2013). How mistimed and unwanted pregnancies affect timing of antenatal care initiation in three districts in Tanzania. BMC Pregnancy Childbirth.

